# Cybersecurity Risk Management Framework for Blockchain Identity Management Systems in Health IoT

**DOI:** 10.3390/s23010218

**Published:** 2022-12-25

**Authors:** Bandar Alamri, Katie Crowley, Ita Richardson

**Affiliations:** 1 Department of Computer Science and Information Systems (CSIS), University of Limerick, Limerick V94 T9PX, Ireland; 2 Lero—The Science Foundation Ireland Research Centre for Software, University of Limerick, Limerick V94 NYD3, Ireland; 3 Health Research Institute (HRI), University of Limerick, Limerick V94 T9PX, Ireland

**Keywords:** Blockchain, Health IoT, identity management, privacy impact assessment, security risk assessment, security risk management, taxonomy

## Abstract

Blockchain (BC) has recently paved the way for developing Decentralized Identity Management (IdM) systems for different information systems. Researchers widely use it to develop decentralized IdM systems for the Health Internet of Things (HIoT). HIoT is considered a vulnerable system that produces and processes sensitive data. BC-based IdM systems have the potential to be more secure and privacy-aware than centralized IdM systems. However, many studies have shown potential security risks to using BC. A Systematic Literature Review (SLR) conducted by the authors on BC-based IdM systems in HIoT systems showed a lack of comprehensive security and risk management frameworks for BC-based IdM systems in HIoT. Conducting a further SLR focusing on risk management and supplemented by Grey Literature (GL), in this paper, a security taxonomy, security framework, and cybersecurity risk management framework for the HIoT BC-IdM systems are identified and proposed. The cybersecurity risk management framework will significantly assist developers, researchers, and organizations in developing a secure BC-based IdM to ensure HIoT users’ data privacy and security.

## 1. Introduction

Blockchain (BC) technology is broadly used for proposing identity management (IdM) system solutions in different domains. Many BC-based IdM system studies relate to Health IoT (HIoT) applications. These solutions aim to provide decentralized IdM systems in HIoT applications so that patients can have control over their identities and data. As IoT is at the centre of these solutions, the security of data derived from them must be considered in IdM systems. Even though BC-IdM systems are attracting attention, one of the main types of barriers to adoption are security and privacy barriers [1]. Thus, the development of a comprehensive security risk management framework will play a pivotal role in assisting researchers in developing secure BC-IdM systems, which, as a result, will encourage acceptance of this type of solution.

The role of security risk management in information systems is to identify assets, security threats, and vulnerabilities that present issues to managing security risks in a controlled and systematic way. These assets can include hardware, software, and networks. There are specific security requirements to protect data assets in these systems. These include integrity, confidentiality, availability, accountability, authenticity, and non-repudiation. A security risk assessment is a crucial step in the risk management process and is vital to expose and mitigate risks [2].

There are several cybersecurity risk assessment standards and frameworks for conducting risk assessments in organizations, such as NIST 800-30 and ISO 27005. These standards provide the general and main steps to conduct security risk assessments, mainly focusing on the security aspect and assuming that every organization should use their frameworks to make a risk management plan. However, some technology paradigms are designed to have data centres in different locations, such as cloud computing and BC technology. These technology characteristics encouraged the development of security risk management frameworks for applications based on these emerging and distributed technologies. For example, Albakari et al. [3] proposed a novel security risk management framework for cloud computing-based applications. Moreover, the privacy aspect should be central to security risk assessments for IdM systems, as the IdM system’s primary goal is to preserve user identity privacy. The EU General Data Protection Regulation (GDPR) mandates applying the Privacy by Design concept and conducting a Privacy Impact Assessment (PIA) in any security risk assessment process.

The contribution of our previous paper [4] was to review the BC-IdM systems in HIoT. In that study, we reviewed 24 studies that proposed BC-IdM solutions in HIoT applications and, as a result, identified the architecture of BC-IdM systems in HIoT, covered security and privacy concerns, and identified the need to develop a comprehensive cybersecurity risk management framework and conduct security risk assessments for BC-based IdM systems in HIoT. Therefore, in this paper, we reviewed 106 studies covering security risks in HIoT, IdM systems, and BC technology, which comprise the HIoT BC-IdM system. The contributions in this paper are as follows:

- Presenting a Systematic Literature Review (SLR) on security risks conducted on HIoT, IdM, and BC;
- Proposing a novel security taxonomy and a comprehensive security framework for HIoT BC-IdM;
- Developing of a novel cybersecurity risk management framework for HIoT BC-IdM;
- Comparing the proposed framework with other frameworks.

The structure of the rest of this paper is as follows: Section 2 covers the background related to the research problem, Section 3 addresses the research methodology, Section 4 addresses the literature review, Section 5 gives a general overview and analysis of the results, Section 6 describes the security taxonomy for HIoT BC-IdM, Section 7 shows the HIoT BC-IdM security framework after mapping data from the identified taxonomy to the HIoT BC-IdM system architecture, Section 8 describes the security risk management framework, Section 9 presents the study limitations and future work, and Section 10 concludes the paper.

## 2. Background

### 2.1. Blockchain-Based IdM

Blockchain is defined as a decentralized database distributed between different participant nodes. It is broadly used for leveraging Self-Sovereign Identity (SSI) management systems, i.e., BC-based IdM systems. The most common BC-IdM applications are uPort, Sovrin, and ShoCard, which attracted an investigation of their security aspects by researchers [5]. Moreover, several studies have also applied Blockchain for developing decentralized IdM systems in HIoT. These studies were reviewed and analysed in [4].

IdM systems are crucial for protecting access to data in HIoT from unauthorized entities. It ensures that only authenticated entities can access data assets based on authorization mechanisms. They control the life cycle of identity in any information system. BC-IdM for HIoT systems is a complicated system that consists of many primary and secondary assets. Our previous work [4] has shown the broad use of BC-based IdM in HIoT, the main technologies and components of such systems, and some of the security and privacy risks that such systems could involve. The main architecture for BC-IdM for HIoT systems consists of the user, application, BC, off-chain, connectivity, and HIoT device layers. IdM system functions mainly work in the BC layer with complementary technologies in off-chain and connectivity layers, especially external storage, such as the Interplanetary File System (IPFS), Application Programming Interfaces (APIs), and cloud technologies.

The IdM system is the main security asset of HIoT BC-IdM and is at the core of this study. The high-level architecture proposed by [6] for BC-based IdM systems involves the following layers and components, as shown in Table 1:

HIoT BC-IdM systems’ identities differ from standard systems that do not involve IoT devices. In HIoT systems, the HIoT identity is a vital part of the IdM system; thus, authentication needs to be guaranteed for these identities.

### 2.2. Standards for BC-Based IdM Systems

Organizations such as W3C and Desterilized Identity Foundation (DIF) proposed emerging standards for BC-based IdM systems, which are used by researchers in applications, such as HIoT. Table 2 includes the most common standards [6].

### 2.3. Architecture of BC-IdM in HIoT

The following is a detailed description of the components and technologies in HIoT BC-IdM [4].

- **Users:** users in HIoT BC-IdM are the stakeholders of the system, such as HIoT service providers, patients, physicians, nurses, and emergency staff. Entities in BC-based IAM can be a thing, a person, or an organization, which plays roles in the BC-based IdM process as a requester, issuer, holder, verifier, and relying party. BC-based IdM user definitions and components are shown in Table 3 [6].
- **Application:** Remote health monitoring systems are used in the HIoT, wallets are used for the IdM system, and APIs are used to exchange data between these applications. Every one of these has security requirements and controls to ensure data protection.
- **BC Technology:** The BC network is at the system’s core, where the IdM’s main functions, such as ID registration, provisioning, de-provisioning, and access control, are performed.
- **Off-chain technology:** In BC-based IdM, there is usually a need to use off-chain storage technologies, such as IPFS, CouchDB, and OrbitDB, to offload data from BC.
- **Connectivity technology:** HIoT BC-IdM-comprised communication protocols, gateways, and technologies used between the system stakeholders and assets, such as HTTP, MQTT, CoAP, and cloud technologies.
- **HIoT device:** Many HIoT device types are used in HIoT systems, which can be classified as either well-being, diagnosis, prognostic, or assistive HIoT devices.

To build a reliable HIoT BC-IdM system, security risks must be considered and managed systematically. Therefore, according to recommendations by [6] about the need for risk management in BC-IdM, as well as findings from our previous study [4], security risk management is needed for HIoT BC-IdM systems.

### 2.4. Security Risk Frameworks

Several risk management standards are designed by organizations, such as NIST and ISO, which are used to conduct security risk assessments and manage risks in organizations and information systems. Risk assessment is a fundamental part of any risk management framework. ISO initially released ISO31000, the *Risk management—principles and guidelines. International Organization for Standardization* [7] in 2009, which was superseded by ISO31000-2018 [8]. Furthermore, ISO released the ISO27000 family of standards, namely the Information Security Management System (ISMS) standards. One of them is ISO27001, which specifies the ISMS requirements, and another is ISO27005, a standard to manage security risks, including security risk assessment processes [9]. On the other hand, NIST published the special publication 800-30 in 2002, *Risk Management Guide for Information Technology Systems*, which was superseded by the *Guide for Conducting Risk Assessment* [10] in 2012, and published the 800-39 *Managing Information Security Risks* [11] in 2011. Among the common frameworks used by organizations and researchers are the NIST-revised SP800-30 and ISO27005 security risk standards [12].

#### 2.4.1. ISO27005 and Related Standards

ISO 27005 [13] is a standard released by the ISO, which is used to manage security risks. It involves three main phases: (1) Risk Identification, which includes assets identification, threats identification, existing controls identification, vulnerabilities identification, and consequences identification; (2) Risk Analysis, which includes risk analysis methodology assignment, assessment of consequences, incident likelihood assessment, and determination of the level of risk; and (3) Risk Evaluation, which defines how to conduct a systematic security risk assessment. It is recommended in ISO27005 to follow the instructions in ISO27001, which include the ISMS security requirements and guidelines to protect data assets, and to apply the information security controls guidelines from ISO27002. For example, in ISO27002 [14], under Section 9 and Section 10, access-control and cryptography guidelines to build secure IdM systems are explained in detail.

#### 2.4.2. NIST 800-30 and Related Standards

NIST 800-30 [10] is another standard used to assess security risk assessment by NIST. Risk assessment involves four main phases: (1) Preparing for the assessment process; (2) conducting the risk assessment, which includes threat source and event identification, vulnerabilities and stimulus identification, likelihood determination, identifying the impact level, and risk determination; (3) communicating the results; and (4) maintaining the assessment process. According to NIST 800-30, there are related standards that need to be applied when applying the standard, such as the managing information security risk (NIST SP 800-39) [11], a guide for applying the risk management framework to information systems (NIST 800-37) [15], security controls for federal information systems and organizations (NIST 800-53) [16], and a guide for assessing the security controls for federal information systems and organizations (NIST 800-53A) [17].

In order to develop a cybersecurity risk management framework for HIoT BC-IdM systems, we used the previous general security risk assessment and management standards and frameworks from related studies, following the research methodology. This work has three main research questions, as follows:

- RQ 1: What are the security requirements, standards, and risks in HIoT BC-IdM?
- RQ 2: What are the components of the proposed cybersecurity risk frameworks in BC, IdM, and HIoT?
- RQ 3: How can a cybersecurity risk management framework for BC-IdM in HIoT ensure security and privacy be developed?

To answer these questions, the authors conducted a Systematic Literature Review (SLR) and a Grey Literature (GL) review on HIoT, BC, and IdM systems to identify security risks, regulations, and standards, as explained in the following section.

## 3. Methodology

**Figure 1:**
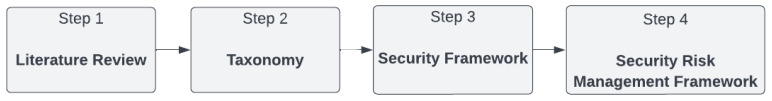
The research methodology phases: (**Step 1**) conduct a literature review. (**Step 2**); taxonomy design.; (**Step 3**) map the data from the taxonomy to the HIoT BC-IdM system.; (**Step 4**) develop the cybersecurity risk management framework.

Four main phases need to be conducted to achieve the goals of this paper, as shown in Figure 1, as follows.

### 3.1. Literature Review

The LR was divided into two parts, a SLR on HIoT, IdM, and BC, following the guidelines to conduct an SLR in software engineering by [18], and a GL on the standards and regulations related to HIoT, IdM, and BC. According to [4], the HIoT BC-based IdM systems involve three main assets, i.e., HIoT, IdM, and BC technologies. Therefore, in order to cover all relevant studies, the related studies to the security of main assets in HIoT BC-IdM should be reviewed. Moreover, as BC is an emerging technology, a GL on the security risk of HIoT BC-IdM was conducted.

### 3.2. Security Taxonomy

The taxonomy was developed following guidelines outlined in [19]. According to Nickerson et al. (2013), “Taxonomies play an important role in research and management because the classification of objects helps researchers and practitioners understand and analyse complex domains”. The purpose of developing a taxonomy plays a huge role in shaping it. The ultimate look of the taxonomy should be based on the eventual user needs. Seven steps are followed to develop the taxonomy in this work, as follows: *Step 1*, meta-characteristics for the taxonomy objects and dimensions were identified in this step. The meta-characteristics for our taxonomy were the components of security risk management in HIoT BC-IdM systems. The maximum number of dimensions was not agreed upon when developing the taxonomy.; *Step 2*, ending conditions were identified in this step. Ending conditions are the benchmark we used to evaluate the completeness of such a taxonomy. They are either subjective or objective; *Step 3*, in this step, the approach to develop the taxonomy was decided. An empirical-to-conceptual approach was chosen and used in this study, in contrast to the conceptual-to-empirical approach, which starts with conceptualizing new characteristics and dimensions. Using the chosen approach, we started with identifying the objects of the three general main assets (HIoT, BC, and IdM), and gradually identified the rest of the dimensions and characteristics; *Step 4*, in this step, we started identifying the subset of the objects of the taxonomy. *Step 5 and 6*, in these two steps, common characteristics for the objects and dimensions were identified, and objects were grouped accordingly. Finally, in *Step 7*, the ending conditions were tested and met. Every developed taxonomy should be concise, comprehensive, robust, explanatory, and extendable. These are the subjective conditions that were met at the end of the taxonomy development process.

### 3.3. Security Framework

In this phase, a comprehensive security framework for HIoT BC-IdM was developed. The architecture identified from our previous SLR study [4] was used in this phase. The security requirements, threats, vulnerabilities, controls, and countermeasures from Step 2 (i.e., taxonomy development) were mapped into it. This framework is an important source, which can be used to conduct cybersecurity risk assessment, threat modelling, and cybersecurity risk management processes for HIoT BC-IdM systems.

### 3.4. Security Risk Management Development

Based on the SLR and GL from this study, which resulted in the development of taxonomy, security and privacy management and assessment frameworks were analysed, which contributed to the design and development of the security risk management framework for HIoT BC-IdM. In addition to that, the common cybersecurity risk assessment and management standards, such as NIST 800-30 and ISO27005 and their related standards, and the proposed frameworks by some researchers in different assets, were used to develop our framework.

## 4. Literature Review

BC is an emerging technology. Thus, to obtain a comprehensive picture of the security of HIoT BC-IdM, we needed to simultaneously use a SLR and GL to cover security requirements, risks, and standards. We also wanted to ensure that we included standards that might not be covered in the literature. This approach was influenced by studies that conducted both SLR and GL, such as [12,20], which identified significant advantages.

Firstly, we separately conducted a SLR on three main assets in the targeted system, i.e., HIoT, IdM, and BC, as every asset had particular security considerations. Initially, the research keywords and their synonyms were identified. As we are targeting security aspects, including privacy, we decided not only to use the “Security” keyword but to also add the “Privacy” keyword to the search process. Furthermore, “Risk” was identified as a keyword, as it was at the core of our research. Moreover, “Health” and “Medical” were identified to be alternative keywords, and we used both. “IoT” was used as an alternative to the “Internet of Things”; thus, both were added to the search process. Finally, we used “Blockchain” as the main keyword and “Distributed Ledger” as an alternate keyword to it.

**Figure 2:**
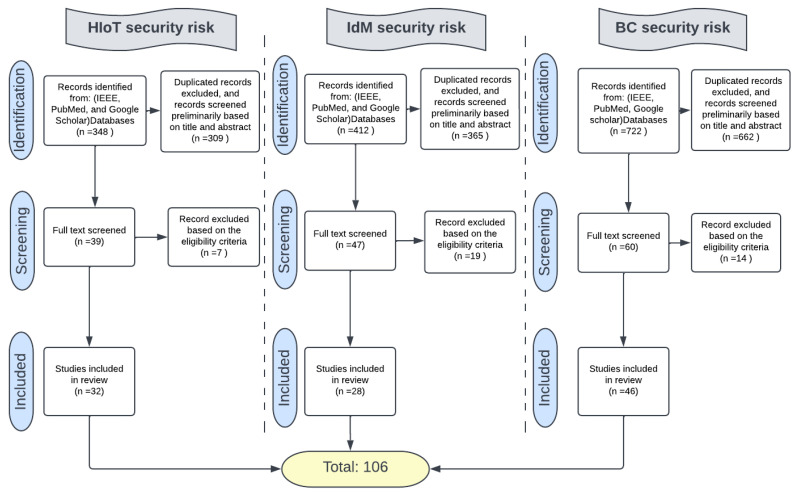
The article selection steps.

We used the strings outlined in Table 4 to extract the relevant studies from our three chosen electronic databases. As the research is interdisciplinary, Computer Science- and Health Informatics-focused databases were reviewed (IEEE explore and PubMed). In addition, Google Scholar was reviewed to supplement these two databases. Figure 2 shows the systematic approach used in order to select the final list of selected papers in the three main assets. Selected articles were tested against an eligibility criterion to ensure that all chosen studies were eligible for the review study. Table 5 shows the inclusion and exclusion criteria used for this purpose. The total number of reviewed studies was 106. There were 32 HIoT studies, 28 IdM studies, and 46 BC studies. A list of the final studies and their contributions can be found in Appendix A.

Secondly, to augment the results from the SLR, we conducted a GL (following the approach used by [12]), where we needed only to use main keywords and specify the targeted literature. We targeted standards and regulations concerning the security and privacy of HIoT, BC, and IdM. The main targeted sources were international standards, regulations, and reports covering the privacy and security risks of the three main targeted systems that make up the HIoT BC-IdM systems. We used main keywords and the inclusion and exclusion criteria shown in Table 6 and Table 7.

## 5. Results

**Figure 3:**
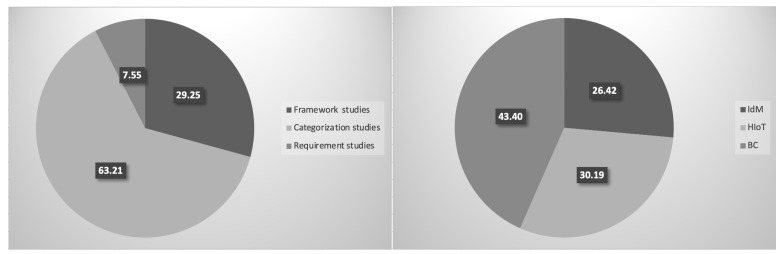
The percentages of the study classifications are based on the covered assets and the main contributions.

All reviewed studies covered security risks in one of the main three assets, namely HIoT, IdM, or BC. However, they can be classified according to their main contributions into three main groups: (1) framework studies focused on security risk management, risk assessment, risk analysis, and threat modelling; (2) studies focused on requirements (e.g., security, privacy, functional, and trust) and controls; and (3) studies categorizing risks, regulations, standards, risk factors, and solutions/countermeasures. Figure 3 shows the percentages of the study classifications based on the covered assets and the main contributions, where framework studies covered 29.25%, categorization studies covered 63.21%, and requirement studies covered 7.55%. Among these studies, 26.42% focused on IdM assets, 30.19% focused on HIoT assets, and 43.40% focused on BC assets.

The majority of the requirements, controls, risk assessments, and management frameworks were derived by researchers and refer to international and national regulations and standards. Several standards and regulations were found in the literature. Some of them were outdated [21] and have been replaced with new versions, such as the British Security Standard BS7799 [22], which was replaced by ISO/IEC risk assessment family standards, such as ISO/IEC27005. Table 8 presents the identified general standards and regulations relating to HIoT, BC, and IdM security risks that are in use. Those that could not be derived from the SLR were derived via GL.

Aside from classifying standards based on their document type (guide/best practice/standard/regulation), they can also be classified based on their purpose (control, risk assessment, risk management, requirements, data protection, etc.), assets (HIoT, IdM, BC-IdM, BC, etc.), or security or privacy aspects. Based on the analysis, the standards can be categorized into four categories according to assets and the scope into general security standards that should be considered in any information systems, such as HIoT, BC, and IdM. General security standards are divided into security, privacy, and data protection standards and regulations. Every standard related to HIoT assets is categorized under HIoT standards, such as medical devices, IoT, cloud, health data, and health software standards. Several standards have been identified regarding IdM identification, authentication and authorization. Lastly, general BC standards and several BC-IdM standards are identified, covering access control, key management, and decentralized identity standards in BC-IdM systems.

Security and privacy attracted the most attention among researchers. Although governmental organizations mandate cybersecurity and data protection laws to protect and preserve health data and patient data, there are still breaches. A study conducted in the USA showed that there is still a lack of work incorporating cybersecurity by design in HIoT to preserve patients’ data [78].

Among the reviewed regulations and laws, a clear difference in dealing with HIoT device privacy is identified. There should be a unified agreement in dealing with the privacy of HIoT [29], not least when emerging technologies, such as BC, are used in it. For instance, GDPR mandates security and Privacy by Design (PBD) [79], which as a result, requires reliable PIA and security risk assessments. The FDA in the USA has regulations in force to ensure all medical devices are registered in their database through the FDA Unified Registration and Listing System [43]. Several studies covered safety as well as security and privacy. According to these studies, safety should be part of any security risk management process of HIoT implementation and maintenance. The objective of HIoT integrity requires protecting patients’ safety [46]. The EU Medical Device Regulation (MDR) mandates considering the safety of HIoT users’ who might be in danger because of a system fault [80]. HIoT user safety is a priority, and there should a compromise between safety, privacy, and security, especially in emergency situations [45].

Several studies identified a lack of standards concerning BC. However, a considerable number of studies conducted risk assessments on BC in general, permissioned BC, or specific BC technologies, such as Ethereum and Hyperledger Fabric. Some focused on a specific aspect of Ethereum or Hyperledger Fabric BC, such as SCs. A number of studies addressed BC-IdM systems specifically, but without conducting/proposing comprehensive risk assessment/management solutions. They covered some of the assets in BC-IdM, such as DID, VC, and DID documents. None of these studies covered BC-IdM for HIoT or any other health or medical applications.

## 6. Taxonomy

Classification science is used in different domains to better understand complicated issues. Computer science and information systems are among the domains that apply taxonomy science [19]. The need for a taxonomy increases when an emerging technology, such as BC, becomes widely adopted. Twenty-nine percent of the reviewed studies, as shown in Figure 3, proposed taxonomies and categorizations for different aspects of the HIoT BC-IdM. They showed classifications and taxonomies for security risk-related topics, such as BC classifications for adoption barriers where security and privacy are considered as one of the main barriers to adopting BC in electronic health record systems and operation management systems [1,81], such as taxonomies for SSI members [20], risk classifications, attack vectors, risk-contributing factors in IdM systems [82], evaluation metrics, cloud IdM security services [83], consequence categories of IdM cyberattacks [84], the privacy characteristics taxonomy in cloud IdM [85], and risk metrics categorizations [86]. These taxonomies were important sources for developing the taxonomy in this research work.

**Figure 4:**
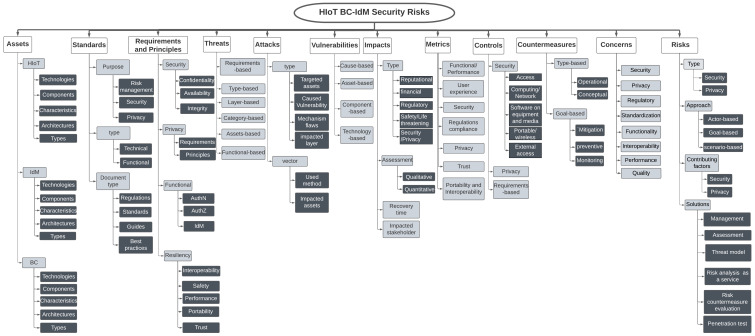
The security risk taxonomy for HIoT BC-IdM systems.

The taxonomy derived from the SLR and GL, and based on the guidelines from [19] (explained in Section 3), is shown in Figure 4. The purpose of developing the taxonomy is to develop a cybersecurity risk management framework for HIoT BC-IdM that allows HIoT cybersecurity researchers and security officers in organizations that use BC-IdM solutions for HIoT to manage cybersecurity risks in a systematic way. These kinds of users are interested in the assets that the system involves, and the cybersecurity risk management framework procedures and components that need to be considered. This purpose limits the characteristics of the taxonomy objects. Thus, we identified three main objects in the targeted system, i.e., HIoT, IdM, and BC assets. Every one of these objects has a number of characteristics, such as security standards, security requirements, threats, vulnerabilities and risks. Moreover, the proposed security risk management frameworks for everyone have other characteristics, such as security control, countermeasures, and metrics, which are used to evaluate the controls and countermeasures. The proposed taxonomy is considered a foundation and can be extended in the future, as the standards and technologies used in BC are evolving. The taxonomy has 12 dimensions that were constructed and identified based on the purpose of the taxonomy, which formed the meta-characteristics (i.e., HIoT BC-IdM assets and security risk management components and procedures). It is explained in detail as follows.

### 6.1. Assets

Assets can be software, hardware, applications, and technologies in any system [11]. HIoT BC-based IdM is a complicated system with three primary assets: BC, IdM, and HIoT systems [4]. Each has several secondary assets, as follows: (1) HIoT’s primary asset consists of HIoT device, network, cloud, and application assets [87]; (2) BC includes on-chain and off-chain technologies, and each has secondary assets [6]; (3) the IdM system itself has secondary assets, such authentication, authorization, and provisioning/de-provisioning operations. In addition, IdM systems have components considered as assets, such as a service provider, identity provider, and a relying party [83], while in BC-IdM systems, there are secondary assets, such as Decentralized Identifiers (DID), Verifiable Credentials (VC), and DID documents [88]. Reviewed studies have presented varied architectures for the aforementioned assets. Some studies used component-based architectures, such as SP, IP, and RP in the IdM systems, or miners, incentivize nodes, etc., in the BC network, or patients, SP, and data consumers in the HIoT system. Whereas some studies developed technologies- or layer-based architectures, such as [89], which are presented for systems such as cloud, communication technologies, and IoT technologies, namely Wireless Personal Area Networks (WPAN). Almost every study showed asset characteristics. They can be issues or features, such as a federation in IdM systems, SSI in BC-IdM, and source constraints in HIoT. Sometimes, BC characteristics become barriers to the adoption of such a technology. For instance, Ref. [81] shows the barriers to using BC in electronic health records, which are caused by some of the BC technology characteristics. According to the study analysis, each of the three main assets has multiple types. HIoT can be wearables [90,91], mHealth [92], WBAN [93] or Medical IoT/miniaturized wireless biomedical devices (MWBDs) [45]. IdM can be conventional, centralized, federated, user-centric, and decentralized [94]. IdM’s two main operations have different models and methods; authorization uses models such as Role-based Access Control (RBAC), Attribute-based Access Control (ABAC), Capacity-based Access Control (CBAC), and Policy-Based Access Control (PBAC) [4,83,88], whereas authentication operations use methods, such as Public Key Infrastructure (PKI), token-based multi-factor authentication, and physically uncloneable functions (PUF) [95]. Several studies proposed Blockchain-based IdM systems for authentication and authorization [4]. Blockchain can be classified as: (1) based on access to policy, whether permissioned, permissionless, or consortium; (2) based on network types, whether private or public; and (3) based on BC platforms and technologies, such as Hyperledger Fabric, Ethereum, and Bitcoin [4].

### 6.2. Standards

Varied standards and regulations are found in the SLR and GL literature. They can be classified based on the document type as regulations [96] (GDPR and DPIA), standards, best practices, reports, or guides, or based on the document purpose (i.e., risk-oriented, control-focused, and security- or privacy-related). They are mainly concentrated on the security of the three primary assets; HIoT, IdM, and BC. Furthermore, they can be classified based on the standard type (functional or technical), where functional standards are mainly a description of the basics in technical standards [97], such as [56], which is a standard that describes the basics of the authentication mechanisms in [57]. It is found from the literature that there are IoT, medical, and IdM system standards and regulations available, which are proposed by international organizations, such as NIST and ISO, in [30,34,35,36,38,44,47,48,49,50,52,55,56,57]; however, when it comes to BC, there is a lack of national and international BC regulations and standards [98]. Additionally, there is a number of technical BC standards proposed by organizations, such as W3C, namely DID and VC [70,76], which are still under development and evolving, and technical reports, such as those in [63,64,65,66,67,68,69,77]. Table 7 shows the regulations and standards derived from the literature.

### 6.3. Requirements and Principles

Security requirements outline the security functionalities that are required in such systems [11]. Requirements (RQMTS) are covered and classified differently in studies. Some studies classified RQMTS based on security aspects, such as confidentiality, availability, integrity, or privacy, which has contextual and content RQMTS, such as anonymity and pseudonymity [99]). In addition to the privacy RQMTS. Studies such as [96,100,101] identified privacy and Privacy by Design principles, such as data quality, accountability, fairness, and data minimization, that need to be in IoT applications. Other studies proposed new classifications, which should be considered with security RQMTS, also known as functional RQMTS. These are the functional RQMTS that the system should perform, such as authorization, authentication, and identity management [33]. Finally, some studies proposed other types of RQMTS, all of which can be categorized under resilience RQMTS (also referred to as non-functional RQMTS), which is proposed by [102], such as safety, interoperability, portability, and reliability. Moreover, trust is a type of RQMTS under resilience RQMTS that should be considered in HIoT BC-IdM systems [103]. They involve procedures performed by IdM systems to ensure trust.

### 6.4. Threats

Security threats are described as events with potential impacts on systems [11]. Threats are classified based on: (1) the impacted security RQMTS (properties) [104], such as integrity [105], availability, privacy, and confidentiality; (2) the threat types, such as Malware and Man in the Middle Attack (MITMA); (3) the impacted architectural layer, such as the HIoT perception layer [95]; (4) the STRIDE (Spoofing, Tampering, Repudiation, Information Disclosure, Denial of Service, and Elevation of privilege) threat categories [12]; (5) impacted assets. Security threats in assets can also be categorized as on-chain threats found in secondary assets, such as SC, consensus, DID, and off-chain threats in secondary assets, such as IPFS [106], cloud servers, sensors, and APIs. SC assets are prevalent in the literature [107,108,109,110,111]; and finally, (6) the functionality defect. According to findings from [4], some HIoT BC-IdM solutions lack some of the main functions of an IdM system, such as identity provisioning/de-provisioning and IdM life-cycle control.

### 6.5. Attacks

Attacks are acts of doing harm to IT assets [10]. According to the analysis of the reviewed studies, they can be classified based on: (1) the targeted assets, such as Smart Contracts [112]; (2) the cause of the vulnerability, such as the double spending vulnerability in BC technologies [113]; (3) the mechanisms’ flaws, such as flaws in consensus mechanisms in BC [114]; and (4) the affected system architecture layers, such as the network layer [115]. In addition, a number of studies identified different attack vectors [74,88,107,114,116]. Attack vectors are another essential classification, and they can either be classified based on the attack method used, such as stealing credentials [116], or based on the targeted asset (hardware or software), such as Smart Contracts [114] and communication protocols [43].

### 6.6. Vulnerabilities

Vulnerabilities are essential parts of every security attack and risk management process. They are defined as weaknesses in the IT assets or the security systems used in these assets [11]. A considerable number of studies covered security vulnerabilities in the HIoT BC-IdM assets. It is noticed that they can be classified based on: (1) the cause of the vulnerabilities [117,118,119], which can be by insider or outsider actors or because of a lack or weakness of security mechanisms [43]; (2) the targeted components; (3) targeted assets; and (4) targeted technologies [43,118].

### 6.7. Impacts

An impact-level evaluation is a step in every risk assessment process. It describes the magnitude of potential damage to IT assets because of security breach activities [10]. There are four classifications found in the literature. Firstly, the impact type, such as regulatory, financial, safety, security, privacy, reputational, and life-threatening impact. Secondly, the impact evaluation approach (i.e., qualitative and quantitative). Thirdly, the impact recovery time. Lastly, the impacted assets or stakeholders (i.e., user, service provider, and manufacturer) [45,120].

### 6.8. Metric Benchmark

It is vital to evaluate the effectiveness of the procedures taken to counter risks by using metric benchmarks to ensure the conducted measures meet the requirements [121]. The metric benchmarks that are covered by studies can be classified into seven groups: (1) functional [122] (in some studies called performance), in which HIoT BC-IdM are evaluated based on the primary functions that the system must perform; (2) security; (3) privacy levels [86,123,124]; (4) trust; (5) user experience [125]; (6) portability and interoperability [71]; and (7) regulation compliance metrics [126]. Moreover, some studies, such as [127], went further and proposed using a tool to evaluate the security countermeasure solutions based on security metrics. Skilled security auditors who are experts in BC-based applications are vital in the security risk management process [128].

### 6.9. Controls

Security controls are measures prescribed to meet and protect security requirements and IT assets [11]. They are mainly derived from standards and regulations and are based on system security requirements [7]. According to the literature, they can be divided into security and privacy controls [16]. Security controls are divided into access, computing and networking, software controls, hardware controls, and external controls [12]. Privacy controls can be classified as contextual privacy controls and content controls [99]. Furthermore, some studies classified controls based on the security RQMTS, such as security controls for integrity.

### 6.10. Countermeasures

Countermeasure techniques are technical measures used to mitigate, prevent, or control security risks. They can be divided based on the literature in two main ways. Firstly, based on the type, they can be either operational countermeasures for privacy and security (i.e., by design solutions) that should satisfy the minimum functions of the IdM system and security/privacy requirements [99,104], or conceptual countermeasures, where countermeasure techniques are discussed theoretically [129]. Secondly, based on the goal of the countermeasures, such as mitigation countermeasures, which target specific security/privacy risks, preventive countermeasures, or monitoring countermeasure solutions.

### 6.11. Concerns

In addition to the concerns and constraints regarding HIoT systems [79,130], several concerns were identified in a number of the reviewed studies concerning security, privacy, regulations, standardization, functionality, quality, performance, and interoperability in BC-based applications [1,71,81,98,122,125,131]. Considering these concerns is vital to building reliable BC-IdM systems for HIoT.

### 6.12. Risk

Security risks arise when unauthorized access to IT assets happens [11]. There are two types of risks, i.e., privacy risks and security risks [132]. Three approaches are used to analyse risks, i.e., actor-, goal-, or scenario-based approaches [133]. The type of risk-contributing factor is another vital classification [82,134]. The risk-contributing factors are classified as privacy- and security-contributing factors. Finally, six types of risk solutions are identified from the literature: (1) novel security risk management frameworks, (2) security risk assessment/risk analysis based on general risk assessment standards, (3) threat models, (4) risk analysis tools as services (static [111] or dynamic [107]), (5) solutions proposed to evaluate security risk countermeasures [124,127], and (6) risk penetration testing solutions.

## 7. HIoT BC-IdM System Security Framework

**Figure 5:**
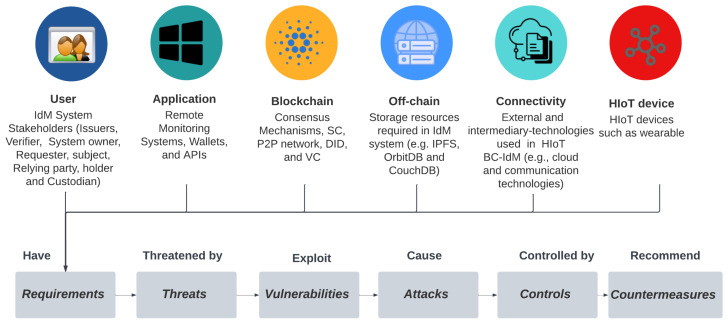
HIoT BC-IdM system security framework.

Several reviewed studies identified the security and privacy threats of HIoT and mapped them to a layered architecture for HIoT; however, none of them covered all the three main assets in HIoT BC-IdM. For instance, [132] mapped the security threats and attacks to the perception, network, middleware, application, and business layers. Such works use the layered architecture that they use in the first place. In this section, we map the identified threats from the literature for the HIoT, BC, and IdM into the layered architecture that we identified in our previous work [4], giving an initial comprehensive security overview. Figure 5 shows the main aspects of HIoT BC-IdM systems (i.e., Assets, Requirements, Threats, Vulnerabilities, Attacks, Controls, and Countermeasures). This is further expanded in Table 9. All security aspects, such as *A*ssets/*C*omponents, are detailed for every system layer, such as *U*ser. Note: threat categories are enclosed using round brackets under the threat aspect in Table 9.

Selected studies were reviewed and analysed, which resulted in developing the security risk taxonomy for HIoT BC-IdM using the guidelines from [19]. Data from the taxonomy that is described in Section 6 were an input for a comprehensive security framework, which is explained in this section, and a cybersecurity risk management framework, which is explained in detail in Section 8. The contributions from the 106 studies are summarized in Appendix A.

## 8. Risk Management Framework

The majority of the reviewed security risk solutions are based on general security risk management frameworks and standards, such as ISO 27005 and NIST 800-30. Thirty-one studies (29.25%) among the reviewed studies conducted/proposed security risk assessment/management solutions in one or more of the main assets in HIoT BC-IdM systems. A comparison between them is conducted to investigate their contributions, the standards applied, and weaknesses and strengths compared with the proposed framework. Table 10 shows a summary of the comparison between these studies. These studies can be classified into security risk studies that propose security risk management frameworks, or studies that conducted a risk assessment, risk analysis, threat modelling, security risk evaluation, and security risk penetration in the three main assets (HIoT, IdM, and BC). HIoT studies either studied HIoT/MIoT generally or focused on one of the HIoT branches, such as wearables [90,91], WBAN [93], mHealth [92], or miniaturized wireless biomedical devices (MWBDs) [45].

General security risk management approaches developed by FDA, ISO, and NIST are too general to be applied to HIoT, especially when it involves emerging technology, such as BC. There are a number of considerations that should be taken into account, such as patients’ safety when such systems process patients’ data. HIoT user safety might interfere with security and privacy; however, security risk management must have a unified assessment process, including security, safety, and privacy [43]. Risk assessment in the HIoT domain lacks a comprehensive risk management approach, not least when it deals directly with access to patients’ data and incorporates BC technologies [78].

**Figure 6:**
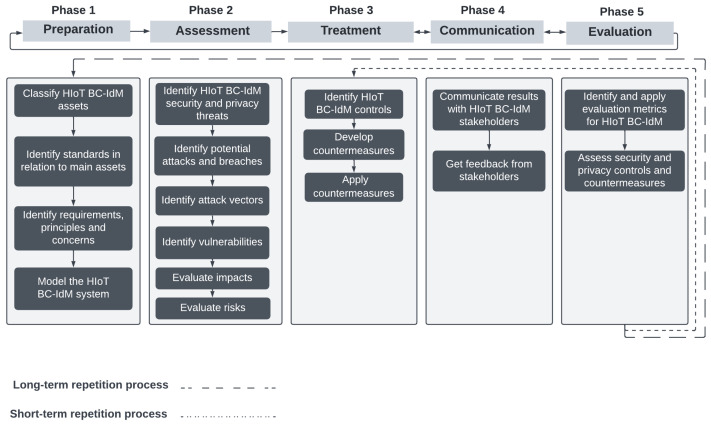
HIoT BC-IdM cybersecurity risk management framework.

To tackle these issues, we propose a comprehensive security risk management for HIoT BC-Based IdM systems, as shown in Figure 6. The proposed security framework for the HIoT BC-IdM system is influenced by three main sources: *First*, general risk assessment frameworks, such as ISO 31000, ISO 27005, and NIST 800-30; *second*, risk management and assessment frameworks that are proposed by some of the reviewed studies for HIoT, IdM, and BC, as shown in Table 10; and *third*, standard and regulation recommendations, such as GDPR, PIA, and security control assessments [17]. For example, EU GDPR requires a data protection impact assessment (DPIA) to mitigate risks to data-subject privacy. The application of DPIA in HIoT BC IdM systems is vital, as previous studies show that there are security threats to identity privacy.

Security risk management in this work is comprehensive and covers security and privacy assessment aspects. Every security risk management process should be based on regulations that are developed by specialist organizations, requirements that are derived from regulations and standards, security controls that are based on requirements, countermeasures that are based on controls, and control assessments derived from regulations and best practices. Countermeasures to mitigate or to stop the risks should be evaluated using metrics that meet system needs and the required functionalities. Moreover, recommendations from experts should be considered to build a reliable IdM system. IdM systems should be designed accurately, and the technologies used in them should be used securely, as their vulnerabilities can be exploited, which might result in user data breaches. Thus, there should be a strong and uncomplicated authentication mechanism to allow users to detect attack activities, such as spoofing attacks, and security and Privacy by Design should be built with the anticipation that attacks are going to happen [135]. Moreover, concerns around used technologies, such as BC, should be identified and dealt with appropriately in order to protect HIoT user security, privacy, and safety.

This framework is developed to be comprehensive and detailed to cover the main phases and sub-steps in security risk management. Therefore, we propose five main phases, each with a number of sub-steps, with each step linked to the previous and following steps, as shown in Figure 6. The steps should be repeated in two different ways. First, a long-term repetition process, in which all the five main steps are applied after a gradual period of time, which can be decided by the organization that owns the system (e.g., annually). Second, a short-term repetition process happens at the end of every time the whole risk management process is conducted. It covers phases 3, 4 and 5. The purpose of this short repetition is to ensure the taken controls and countermeasures are adequate and meet the decided evaluation metrics. The output from the last sub-step in every main phase will be an input for the first sub-step in the following main phase, as explained below in detail.

### 8.1. Preparation

In this phase, four sub-steps should be conducted. *The first step* is to classify assets in the HIoT BC-IdM system, namely HIoT, IdM, and BC. It should be noted that each of these main assets has secondary assets, for example, BC has SCs, DID, VC, consensus mechanisms, and internal and external DBs, such as CouchDB and LevelDB. Table 9 will help identify the assets. The security risks relating to emerging BC-IdM standards, namely DID and VC and their components, such as DID documents, are well covered in the literature, as shown in Appendix A. *The second step* is to identify standards and regulations concerning the system. Standards and regulations related to HIoT BC-IdM systems are identified and summarized in Table 8. Some are national data protection regulations, such as GDPR, PIPA, PDPA, HIPAA, and SHIEP, where data protection activities are mandated, such as PIA, which is mandated by GDPR. Some are technical and functional security standards developed to give best practices in developing security mechanisms. This phase must identify the HIoT BC-IdM security standards and regulations that should be complied with. For example, IoT security standards, such as IEEE 802.15.6 protocols, which are recommended for HIoT networks, and national and international regulations, such as those published by HIPAA and FDA, need to be applied. Furthermore, some standards relate to compliance with identity management and access-control systems. For example, the ISO27002 standard focuses on guidelines about information security controls. Section 9 and Section 10 in ISO27002 cover access-control and cryptography guidelines. Medical device standards, such as ISO 13485:201, also need to be considered. *The third step* is to identify security, functional, and resiliency requirements, privacy requirements and principles, and risk factors and concerns to build a concrete HIoT BC-IdM. Various HIoT BC-IdM used technologies that have different components, thus having different security requirements and privacy principles. Therefore, they should be identified. Moreover, several concerns are addressed in the reviewed studies, particularly concerning BC-IdM technologies [71,125,131]. All these concerns and challenges should be identified to be dealt with appropriately. Finally, *the fourth step*, is to model the HIoT BC-IdM system, showing the data flow of the system. A data flow diagram (DFD) is used to show how data flows between assets, stakeholders, and trust boundaries (i.e., a component of the DFD used to describe when data flow changes from one level to another level within the system).

### 8.2. Assessment

This phase is at the core of the security risk management framework and consists of six sub-steps. *Step one* is to identify the HIoT BC-IdM threats using the DFD from Phase 1, which limits the scope for all threats. The STRIDE threat-modelling approach can be used to identify security threats, and the LINDDUN approach can be used to identify privacy threats in this step. Security and privacy threat identification is vital for the following phases. Security threats, including privacy threats, are identified in this phase. *Step two* is to identify the potential attacks and breaches. *Step three* involves identifying attack vectors. *Step four* requires the identification of vulnerabilities. Vulnerabilities identification is a sophisticated phase in security risk assessment where all security weaknesses for the HIoT BC-IdM systems are discovered. *Step five* involves the evaluation of impacts using qualitative or quantitative methods. An analysis of the likelihood of vulnerabilities being exploited and the expected risk impact from threats are covered in this step. *Step six* evaluates the risks, preparing to identify the most appropriate security controls and countermeasure mechanisms.

### 8.3. Treatment

This phase involves three sub-steps. *Step one* is to identify the controls of the HIoT BC-IdM system. Identifying privacy and security controls is a vital step in order to build reliable countermeasure solutions. They are classified as explained in the taxonomy to security and privacy. The countermeasures are built and applied based on the controls, which include a plan for solutions to mitigate the estimated risks. *In step two*, the countermeasure solutions are developed based on the controls, taking into account security, privacy, the safety of HIoT users, and the BC-IdM functionality needs. *The last step* in this phase is to apply the developed solutions.

### 8.4. Communication

It is vital to seek feedback from other stakeholders. Communication should be an active activity, in which HIoT BC-IdM system users and other stakeholders, such as developers and service providers, can give feedback and take part in reviewing the solutions [125]. As well as keeping the stakeholders aware of the system’s security risks, communications with HIoT users are vital to ensure the cybersecurity of these devices. Some reviewed studies conducted interviews with HIoT users, such as [136], which showed the essential need for adding a communication phase in cybersecurity risk management in HIoT applications. This phase involves two sub-steps, namely *the first step*, which communicates the processes’ results with the system stakeholders, and *the second step*, which seeks feedback from them to consider in the evaluation phase, which is the following and last main phase. Decision making regarding developing the countermeasures requires consultation from stakeholders, such as HIoT users and experts.

### 8.5. Evaluation

The final phase ensures all countermeasure solutions apply the security controls and meet the requirements of the HIoT BC-IdM system. This phase includes an evaluation process of the procedures taken. *Step One* in this phase involves the identification of metrics to evaluate requirements and controls, and also the methodology to evaluate solutions. The previously conducted SLR [4] showed that the proposed HIoT BC-IdM solutions lack primary functional requirements, such as identity management life-cycle control. Thus, this step is vital. Using a systematically built framework in this phase, such as the ISA framework by [124], is vital to evaluate and ensure reliable countermeasure solutions. *Step Two* is to assess the security and privacy controls. Standards that are recommended in [17] outline a methodology to assess security and privacy controls in the whole life cycle of the system. This framework proposes regular long-term repetition processes (outlined by the broken line) in Figure 6 to the whole security risk management process, as the core technology (i.e., BC) is an emerging and evolving technology and because regulations, technical standards, and new platforms around it are constantly evolving. Changes in regulations, such as GDPR and HIPAA, and technical standards, such as DID, are expected to change to meet BC-requirement changes; thus, they should be regularly reviewed for compliance. In addition, BC-based IdM system standards, such as DID and VC, are vulnerable to privacy and security flaws, and alternative standards or tested standards might be used instead [131]. Furthermore, a short-term repetition process is proposed, in which security and privacy controls and countermeasures are reviewed and evaluated whenever the risk assessment process has taken place. Therefore, in case a weakness is found, critical feedback is given from stakeholders regarding controls and countermeasures that do not meet the evaluation metrics; then, the changes are implemented in this iterative process (outlined by the dotted line) in Figure 6.

To apply the proposed security risk management framework on HIoT BC-IdM systems, it is recommended to form a technical team consisting of members from the healthcare setting, HIoT users, SP members, BC experts, IdM experts, and security risk assessment experts, such as data protection officers (DPO). The team members should work together throughout the testing process of the system, as recommended by [93]. They should ensure the security and privacy of HIoT users’ data and identity, as well as HIoT user safety, which might be at risk because of HIoT security breaches [46].

**Table 10:** A comparison between HIoT BC-IdM cybersecurity risk framework studies.

Authors	Contributions	Strengths	Weaknesses
[S1] Sepczuk and Kotulski [22]	Risk assessment as a service for IdM authentication, applies ISO/IEC27005.	Covers authentication process in IdM systems.	Does not follow risk management standards.
[S2] Wang et al. [31]	Risk assessment for BC applications within China, follows the Chinese Classified Protection Cybersecurity (CPC) law.	Based on national standards. It covers Bitcoin, Ethereum, and Hyperledger Fabric BCs and gives evaluation metrics and controls for P2P network, consensus, Distributed Ledger, and contract layers.	It lacks main components of risk management.
[S3] Kim et al. [32]	Risk analysis for DID document in the W3C DID technical standards.	Scenario-based risk analysis for DID authentication used to provide Self-Sovereign Identity technologies.	Does not follow risk management standards.
[S4] Vakhter et al. [45]	Threat modelling and risk analysis for HIoT (miniaturized) applies NIST SP 800-30.	Covers HIoT assets with a focus on miniaturized HIoT, and gives risk analysis.	Does not cover BC and IdM assets.
[S5] Schlatt et al. [74]	BC cybersecurity framework for BC.	Covers the relations between stockholders (users, developers, attackers) in BC applications and the BC infrastructure.	Lack of main components of risk management.
[S6] Alzahrani et al. [81]	Assessment model for BC-based electronic health records.	Covers BC-based electronic health records and security and privacy risks.	General assessment does not follow risk management standards.
[S7] Psychoua et al. [90]	Privacy risk assessment for HIoT (wearable).	Covers privacy aspect with a focus on Privacy by Design.	Does not follow risk management standards and does not cover BC and IdM assets.
[S8] Tseng et al. [91]	Risk assessment for HIoT (wearable) using STRIDE and DREAD approaches.	Covers HIoT assets.	Does not follow risk management standards and does not cover BC and IdM assets.
[S9] Cagnazzo et al. [92]	Threat modelling for HIoT (mHealth) using STRIDE and DREAD approaches.	Covers HIoT assets.	Does not follow risk management standards and does not cover BC and IdM assets.
[S10] Paul et al. [93]	Risk management for HIoT applying ISO/IEC 80001-and AAMI TIR57.	Proposes security risk management for HIoT(WBAN) and reviews regulations/standards and security and privacy controls.	Does not cover IdM and BC assets.
[S11] Sheik et al. [94]	Threat modelling for BC-IdM using the STRIDE approach.	Covers BC-IdM.	Does not follow risk management standards and does not cover HIoT assets and emerging BC-IdM standards, such as DID.
[S12] A Shostack [100]	General threat modelling methodology.	Covers Security and Privacy.	It is general and does not support short-term repetition processes.
[S13] Bhardwaj et al. [107]	Dynamic penetration test for SC-based applications. Applies OWASP top 10 vulnerabilities.	Covers BC SC.	Does not follow risk management standards and only focuses on SC assets.
[S14] Lv et al. [111]	Static risk analysis for SCs in Hyperledger Fabric.	Covers SC assets in Hyperledger Fabric.	Does not follow risk management standards and only focus on SC assets.
[S15]Wen et al. [115]	BC cybersecurity framework.	Covers attacks and countermeasures in a BC-layered framework.	It lacks risk management main components.
[S16] Naik et al. [116]	Tree-based risk analysis for BC-IdM (SSI).	Covers BC-IdM components, such as DID, and shows attack vectors.	It does not follow risk management general standards and does cover HIoT assets.
[S17] Konig et al. [117]	Risk analysis for BC.	Presents a BC-layered framework and shows the prerequisites for attacks.	Does not follow risk management standards.
[S18] Alsubaei et al. [118]	Security risk assessment for HIoT (risk assessment as a service (tool) testing 260 attributes), and considers standards, such as HITECH Act, HIPPA, GDPR, PCEHR Act, ISO/iec27018, ISO/IEC 27034, AICPA, FIPS, GSMA, MDD39/42/EEC, MDR2017/745, ISO/IEC80001, ISO14971, ISO13485, ISO/IEC22301, and ISO/IEC27001.	Covers HIoTs.	Does not follow risk management standards and does not cover IdM and BC aspects.
[S19] Wang et al. [124]	Uses Identified Security Attributes (ISA) framework for HIoT.	Covers HIoT assets and gives systematic approach to evaluate security solutions and decision making.	Does not follow risk management standards and does not cover BC and IdM assets.
[S20] Lopatina et al. [137]	Risk assessment for HIoT.	Covers HIoT assets.	Does not follow risk management standards and does not cover BC and IdM assets.
[S21] Mallah et al. [138]	Security risk assessment for BC-based transportation applications. Uses ISO31000 and ISO27005.	Covers BC Assets.	Does not cover HIoT and IdM assets.
[S22] Ruf et al. [139]	Threat modelling for BC-based industrial IoT applications.	Covers BC assets and presents a case study.	Only on-premise threat analysis, does not give details about threat modelling methods, and does not cover HIoT and IdM assets.
[S23] Cha et al. [140]	Security control framework for permissioned BC applications, and uses PCI-DSS, CIS controls, and ISO/IEC27001 and ISO/IEC 27002 standards.	Covers controls in different layers.	Does not cover the main security risk management phases.
[S24] Morganti et al. [141]	Risk assessment for BC technology, which follows NIST SP-800-30.	Covers BC assets.	Covers BC in general but does not cover HIoT and IdM assets.
[S25] Homoliak et al. [142]	Security reference architecture (SRA)-based risk assessment for BC technology, which uses ISO/IEC 15408 standards.	Covers BC nodes (consensus, validating, lightweight), and gives detailed analysis of threats, vulnerabilities, and defences.	Covers BC applications in general.
[S26]Putz and Pernul [143]	Threat modelling for Hyperledger Fabric BC.	Covers BC assets and threat indicators in Hyperledger Fabric BC.	It lacks the main components of security risk management.
[S27] Zhao et al. [144]	Risk analysis for BC technology communications.	Presents a BC-layered framework.	Does not follow risk management standards.
[S28] Wilson et al. [145]	Digital identity security framework for IdM in IoT systems.	A stack model covers privacy in IdM.	Does not follow risk management standards, and does cover HIoT and BC assets.
[S29] Arias-Cabarcos et al. [146]	Risk assessment for IdM, which uses multi-attribute utility theory (MAUT).	Covers IdM physical and digital authentication aspects and gives quantitative evaluation for security and privacy.	Does not follow risk management standards.
[S30] Attaallah et al. [147]	Risk assessment for HIoT.	Covers the security requirements of HIoT.	Does not follow risk management standards, does not cover IdM and BC assets, and lacks details.
[S31] YIN et al. [148]	Security risk management for HIoT, which applies ISO/IEC27005 standards.	Presents a case study in a hospital.	Lacks details and does not cover BC and IdM assets.

## 9. Limitations and Future work

This study includes a systematic review of the literature on the security risks of three systems that comprise the system under study, namely HIoT, IdM, and BC. Following the guidelines of the used search approach, IEEE Explore and PubMed databases were chosen to be reviewed because the study domain is interdisciplinary. In addition, Google Scholar was used to supplement them. This study investigates and develops a unified cybersecurity risk management framework for HIoT BC-IdM, with no emphasis on a specific type of HIoT, in order to provide a general and unified framework. Because using BC for IdM systems in HIoT is a relatively new domain, there is an opportunity to conduct more specific studies in the future on all HIoT types, such as wearables. Furthermore, the security requirements, threats, vulnerabilities, and controls are mapped from the SLR and GL to the HIoT BC-IdM system; however, in order to provide a more detailed study, this work will be followed by a demonstration work in which the proposed security risk management framework will be applied. This study’s findings will be used to inform future efforts to conduct systematic security risk assessments. Furthermore, the proposed security risk management framework will be presented to a group of domain experts, who will evaluate it and its applications using methodologies such as *Delphi*.

## 10. Conclusions

This research work investigated the security and privacy risks of HIoT BC-based IdM systems and proposed a security taxonomy, security framework and a cybersecurity risk management framework for HIoT BC-based IdM systems. In order to answer the three research questions, we developed a research methodology consisting of four main phases. Firstly, SLR and GL reviews were used to collect relevant data. A total of 106 studies were included in the SLR. A GL was used to complement the SLR to ensure standards related to the system assets, such as BC and cloud, are included. Secondly, after listing and analysing the results from the first phase, we proposed a risk security taxonomy which classified the outputs of the studies concerning the security risk management components and procedures in a systematic way. The classified data give a clear and comprehensive overview of the work to date concerning HIoT BC-IdM systems, which address the main components of the proposed cybersecurity risk management framework. Thirdly, we proposed the HIoT BC-IdM security framework by analysing risks, threats, vulnerabilities, requirements, and controls and mapping them from the taxonomy to the layered architecture for the HIoT BC-IdM system. Finally, we developed the security risk management framework by comparing the selected reviewed studies that proposed risk assessment, risk analysis, threat modelling, and risk management in the main assets in HIoT BC-IdM systems and analysing the identified components.

The proposed taxonomy, security, and cybersecurity risk management frameworks are novel and holistic. They are essential in order to develop secure BC-IdM solutions for HIoT. Our previous SLR showed that the proposed HIoT BC-IdM solutions do not follow a comprehensive and systematic security and risk management framework. Our framework will play a significant role in protecting HIoT users’ data by assisting researchers and, as a result, helping to use BC technologies to systematically develop a decentralized IdM system for HIoT.

## Figures and Tables

**Table 1:** The BC-based IdM system layers and components.

Layers	Description
Blockchain	BC technologies are IdM-specific, such as Indy Hyperledger- or Smart Contract-supported platforms, such as Ethereum and Hyperledger Fabric, which are are used to facilitate Public Key Infrastructure (PKI) to ensure data integrity in the IAM system.
Second-layer protocol	Offload solutions for scalability by proposing a top layer, where Smart Contracts and other technologies are used in the IdM system.
Smart Contracts	The majority of BC-based IAM build their logic based on Smart Contracts.
Credential storage methods	Off-chain storage where credentials are stored, as not all BC-based IdM systems provide on-chain storage for credentials.
User-controlled identity wallet	Applications with APIs are used by users to store identifiers, credentials, and their corresponding private keys and allow entities to exchange credentials and presentations with each other.
User-profile data management protocols	An external protocol is used for storing user profile data, such as browsing data, user settings, and transaction history.
Data exchange models	Data exchange models, such as JSON, SAML, XDI, and JWT are used to initiate, verify, and disclose data, such as credentials and representations.
Application libraries and interfaces	APIs and applications allow communication between IdM roles (i.e., requester, issuer, relying party, and verifier).

**Table 2:** The most common modern standards for BC-IdM systems.

Standards	Description
Decentralized Identifiers	W3C develops Decentralized Identifiers (DIDs) to facilitate private channels between entities, eliminating the need for a central registration authority.
Verifiable Credentials and Verifiable Presentations	Verifiable Credentials (VC) and Verifiable Presentations (VP) are standards developed by W3C used to format credentials.
Universal Resolver	It is developed by the Decentralized Identity Foundation (DIF) to retrieve DID documents.
Identity Hubs	Off-chain storage developed by DIF.
DID Auth–RWOT	Authentication framework to unsure DID ownership.

**Table 3:** The BC-based IdM user definitions and components.

Object	Description and Role
Entities	It can be a thing, a person, or an organization with one or more identifiers.
Identifiers	An entity pseudonym or BC address can be associated with one or more credentials.
Credentials	One or more claims are associated with an identifier used to build presentations.
Presentations	Information extracted from credentials.
Document	Metadata about an identifier.
Claim	Subject characteristics are used as part of the credentials.
Custodian	An entity acts as another entity in the BC-based IAM system.
Holder	An entity is holding credentials on behalf of another one.
Issuer	An entity is issuing credentials.
Relying party	An entity is responsible for receiving derived information from the verifier.
Requester	An entity requests subject credentials from the issuer.
Subject	An entity obtains credentials from the issuer.
System owner	System owner.
Verifier	An entity in charge of the presentation verification and validation processes on behalf of the relying party.

**Table 4:** The strings used in the search process.

Targeted Literature	String
Health IoT systems	(Security OR Privacy) AND Risk AND (Health OR Medical) AND (IoT OR Internet of Things)
IdM systems	(Security OR Privacy) AND Risk AND Identity Management
Blockchain technology	(Security OR Privacy) AND Risk AND (Blockchain OR Distributed Ledger)

**Table 5:** The inclusion and exclusion criteria used in the SLR.

Inclusion Criteria	Exclusion Criteria
English-written studies	Studies are written in languages other than English.
Without time-frame restriction	Concept papers.
Open access peer-reviewed studies	Previous work (with no added valuable contributions), when a work has been extended.
Secondary and primary studies conducting/proposing risk analysis management, assessment, or threat modelling.	Primary studies that only propose security solutions other than risk analysis, management, assessment, or threat modelling.
Secondary and primary studies identifying security/privacy risks, standards, requirements and controls.	
HIoT-, IdM-, and BC-focused studies	Studies that cover HIoT as a part of comprehensive health/medical information applications.

**Table 6:** The keywords and inclusion and exclusion criteria used in the search process for GL.

Targeted Literature	Keywords
Health IoT systems	Health IoT, Medical IoT, Security, Privacy, Risk Assessment, Risk Management, Standards, and Regulations.
IdM systems	Digital Identity, Identity Management, Security, Privacy, Risk Assessment, Risk Management, Standards, and Regulations.
Blockchain technology	Blockchain, Distributed Ledger, Security, Privacy, Risk Assessment, Risk Management, Standards, and Regulations.

**Table 7:** The inclusion and exclusion criteria used in the GL.

Inclusion Criteria	Exclusion Criteria
English-written studies.	Studies are written in languages other than English.
Without time-frame restriction.	
International and national regulations, standards, and reports about the targeted literature.	Superseded regulations, standards, and reports.

**Table 8:** The identified standards relating to HIoT, BC, and IdM systems.

Standards	Type	Assets/Scope	Considerations
NIST BC-based IdM [6]	Guide	BC (IdM)	Overview, guidelines, and issues about the Blockchain-based identity management systems.
NIST800-30 [10]	Standards	General	Conducting risk assessment.
NIST800-39 [11]	Standards	General	Managing information security risks.
OWASP [12]	Standards	HIoT (Medical Device)	Security controls include privacy impact assessment, security audit, perimeter defences, network controls, device security controls, and end-user interface controls.
TGA [12]	Guide	HIoT (Medical Device)	The Australian medical device cybersecurity guide, which includes cybersecurity principles and threat and risk assessment processes.
ISO27005 [13]	Standards	General	Information security risk management.
ISO27002 ISO 27002/27001 [14]	Best Practice	General	Information security, cybersecurity, and privacy protection—information security controls.
NIST800-37 [15]	Guide	General	*Risk Management Framework for Information Systems and Organizations: A System Life-Cycle Approach for Security and Privacy*.
NIST 800-53 [16]	Best Practice	General	NIST security and privacy controls.
NIST 800-53A [17]	Standards	General	Assessing security and privacy controls in information systems and organizations.
CIS controls [23]	Best Practice	General	A total of 18 security controls to mitigate security attacks.
PCI-DSS: Payment Card Industry Data Security [24]	Standards	General	It includes a set of requirements, such as maintaining a secure network, customer data protection, vulnerability management, access control, network monitoring, and information security policy.
EU Network and Information Security (NIS) directive [25]	Directive	General	Objectives to ensure security among EU countries.
ISO/IEC 29100 [26]	Standards	General	Privacy framework provides privacy terminologies, defines the actors and their roles in processing personally identifiable information (PII), identifies and describes privacy safeguarding considerations and principles.
ISO/IEC 15408-1 [26]	Standards	General	Evaluation criteria for IT security.
ISO 27018 [26]	Standards	HIoT (Cloud)	International standard for protecting personal identifiable information (PII) in cloud storage.
GDPR [27] and GDPR-DPIA [28]	Regulation	General (Data Protection)	The EU general data protection regulations that emphasize data-subject protection rights. Articles 76, 77, and 35 in GDPR mandate the conducting of a data protection impact assessment (DPIA)(i.e., privacy impact assessment (PIA)) within the security risk assessment.
PIPEDA and SHIEP [29]	Regulation	General (Data Protection)	The Canadian Personal Information Protection Electronic Document Act (PIPEDA) and the Saudi Health Information Exchange Policies (SHIEP). They emphasize data-subject privacy.
IEEE 802.15 [29]	Standards	HIoT (IoT)	Wireless Personal Area Network (WPAN) standards cover security and access control of low-range IoT devices.
ENISA [30]	Report	HIoT (general)	Smart hospitals security and resilience for smart health service and infrastructures.
CPC [31], PIPA [32], PDPA, PA1988 and FIA [33],	Regulation	General (Data Protection)	Chinese Classified Protection of Cybersecurity, Personal Information Protection Act of Korea, Malaysian Personal Data Protection, Australian Privacy Act 1988, and American Freedom of Information Act. They emphasize data-subject privacy.
ISO14971 [34]	Standards	HIoT (Medical Device)	Application of risk management to medical devices.
ISO24971 [35]	Standards	HIoT (Medical Device)	Guidance on the application of ISO 14971 risk management.
ISO80001 [36]	Standards	HIoT (Medical Device)	Application of risk management for IT networks incorporating medical devices.
FDA Cybersecurity in Medical Device [37]	Guide	HIoT (Medical Device)	FDA Pre- and Post-market considerations of cybersecurity in medical devices, threat modelling, and risk management.
IEC 62304 [38]	Best Practice	HIoT (Medical Device)	Medical device software—software life-cycle processes show the security requirements.
AAMI TIR57 [39]	Guide	HIoT (Medical Device)	Principles for medical device security and risk management. Provides guidance on methods to perform information security risk management for a medical device in the context of the safety risk management process required by ISO 14971.
IMDRF [40]	Guide	HIoT–Medical Device	Principles and best practices for medical device cybersecurity.
MITRE rubric [41]	Report	HIoT (Medical Device)	Rubric for applying Common Vulnerability Scoring System (CVSS) to medical devices.
EU Directive 2017/745 and 2017/746 [42]	Regulation	HIoT (Medical Device)	The European Medical Device Regulation (EU MDR): standards of safety, security, and quality of medical devices within the EU.
ICE60601 [43]	Standards	HIoT (Medical Device)	Assessment to guarantee the compliance to EU MDR.
NISTIR 8228 [44]	Standards	HIoT (IoT)	Covers IoT device capabilities, security, privacy considerations, and challenges, as well as recommendations on how to mitigate security risks. Covers three main aspects: device security protection, data security protection, and individual privacy protection.
NIST SP 800-213 [45]	Standards	HIoT (IoT)	IoT device cybersecurity guidance identifies the IoT device cybersecurity requirements.
NIST8200 [46]	Standards	HIoT (IoT)	Interagency report on the status of international cybersecurity standardization for the Internet of Things (IoT). It covers IoT applications, including Health IoT, cybersecurity risks and threats, cybersecurity areas, and standard landscape for IoT cybersecurity.
NISTIR8259 [47]	Standards	HIoT (IoT)	Foundational cybersecurity activities for IoT device manufacturers. Cybersecurity risks related to IoT.
NISTIR8259A [48]	Standards	HIoT (IoT)	Internet of Things (IoT) device cybersecurity capability core baseline, which is a set of device capabilities generally needed to support common cybersecurity controls that protect an organization’s devices, as well as device data, systems, and ecosystems.
ISO/IEC 27400 [49]	Standards	HIoT (IoT)	Cybersecurity–IoT security and privacy guidelines. This guide provides guidelines on the risks, principles, and controls for the security and privacy of Internet of Things (IoT) solutions.
ETSI EN 303645:European Standards [50]	Standards	HIoT (IoT)	*Cybersecurity for Consumer Internet of Things: Baseline Requirements*. It shows the baseline requirements in order to protect IoT user security.
GSMA [51]	Standards	HIoT (IoT)	IoT security guidelines show the IoT models, challenges, privacy considerations, and IoT risks assessment.
HIPAA [52]	Regulations	HIoT (Health Data)	Privacy rules for health data and identifiable health information.
HL7 [53]	Standards	HIoT (Health Data)	Standards to exchange health data in electronic health records.
IEC 81001-5-1 [54]	Best Practice	HIoT (Health Software)	Guidelines on the product life cycle of health software and health IT systems safety, effectiveness, and security.
IEC 82304-1 [55]	Standards	HIoT (Health Software)	ISO standards concerning the safety and security of health software products.
ISO/IEC 9798 part 1 and part 2 [56,57]	Standards	IdM	Entity authentication standards and specifications for mechanisms using authenticated encryption algorithms.
ISO/IEC 29115 [58]	Standards	IdM	Security techniques–entity authentication assurance framework.
NIST800-63-3 [59]	Standards	IdM	Digital identity guidelines. Shows models and digital identity risk management.
EIDAS [60]	Regulation	IdM	EU regulation on electronic identification. eIDAS (electronic identification, authentication and trust services) was legislated to ensure secure cross-border transactions within the EU.
IEEE 2410 SBP [61]	Standards	IdM	Standard for Biometric Privacy (SBP) provides private identity assertion.
ISO/IEC 24760 part 1 and part 2 [62]	Standards	IdM	A framework for identity management.
EU Blockchain Observatory and Forum [63,64,65,66]	Report	BC	Several reports about BC applications and regulations in the healthcare and public services.
ESMA [67]	Report	BC	Report titled “The Distributed Ledger Technology Applied to Securities Markets.” It discusses risks, benefits, and DLT issues.
ISO/TR 23455 [68]	Standards	BC	Blockchain and Distributed Ledger technologies—overview of interactions between Smart Contracts in Blockchain and Distributed Ledger technology systems. It covers different platforms, such as Ethereum, Bitcoin, and Hyperledger Fabric.
NIST IR 8403 [69]	Guide	BC (IdM)	Guidelines of access-control part of BC-IdM systems.
W3C [70]	Standards	BC (IdM)	Decentralized Identifier (DID), Verifiable Credentials (VC), and Verifiable Presentations technical standards by W3C, which facilitate the connection between entities without a central party.
DIDAuth [71]	Standards	BC (IdM)	Authentication framework to unsure the DID ownership.
Decentralized Identity Foundation (DIF) standards. [72]	Standards	BC (IdM)	Identifiers, DID authentication, claims and credentials technical standards for decentralized identity management systems.
Ethereum DID [73]	Standards	BC (IdM)	Ethereum decentralized digital identity technical standards.
ERC-721 [74,75]	Standards	BC (IdM)	Ethereum non-fungible token standards.
DKMS [76]	Standards	BC (IdM)	Decentralized cryptographic key management systems standards.
NISTIR 8301 [77]	Guide	BC (IdM)	Guidelines of tokens in BC-IdM systems.

**Table 9:** Details of the HIoT BC-IdM system security framework.

**Users**	
Assets/Components	Issuers, verifiers, holder custodian, data subject, system owner, holder, relying party, and orderers.
Requirements	Integrity, availability, confidentiality, non-repudiation, anonymity of users, patient-control, fine-grained access control, and authentication of users.
Threats	User device impersonation (spoofing), patient data tampering (tampering), and malicious input (tampering).
Vulnerabilities	Weak password and insider-threat vulnerabilities.
Control and Countermeasures	Authentication/ multi-factor authentication, authorization, and auditing ABE key management.
**Application **	
Assets/Components	Remote monitoring system, personal wallets, and Application Programming Interface (APIs).
Requirements	Integrity, availability, confidentiality, and non-repudiation.
Threats	Insecure APIs (elevation of privilege), unsecured software components (spoofing, tampering, information disclosure, and elevation of privilege), lack of input/output filtering in HIoT and APIs (tampering and information disclosure).
Vulnerabilities	Unsecured interfaces, lack of authentication and authorization, lack of privacy mechanisms, and lacking/weak encryption.
Control and Countermeasures	Logging and access control.
**Blockchain**	
Assets/Components	Peer-to-Peer (P2P) Network, consensus mechanisms, validation nodes, incentives, punishment mechanisms, IdM system, Oracles (a type of software), Smart Contracts (SCs), DID, and VC.
Requirements	integrity, availability, confidentiality, accountability, non-repudiation, privacy, intervenability, unlinkability, transparency, identity data locality, trust, and consistency of transactions.
Threats	Consensus mechanism vulnerabilities, Sybil attack, double-spending threat, Smart Contract/Chaincode threats, replay attack (tampering), quantum threats, 51 attacks (majority attack), Decentralized Identifier (DID) defects, insider threats, and advance persistent threat (APT).
Vulnerabilities	Centralization of control, shared untrusted networks, P2P protocols vulnerabilities, Domain Name System (DNS) and routing protocol vulnerabilities, Ethereum virtual machine vulnerabilities, SC programming language vulnerabilities, dataveillance problems in the DIDs, and forgery attacks on BC network.
Control and Countermeasures	Authentication, input validation, session management, encryption, using quantum-safe cartographic mechanisms, using one of 51 attack prevention techniques, using SC analysis tools, using SC countermeasure analysis tools, secure Membership Service Provider (MSP), strict access, and infeasible service endpoint attributes.
**Off-chain**	
Assets/Components	External DBs and storage, such as IPFS, CouchDB, and LevelDB.
Requirements	Integrity, availability, and confidentiality.
Threats	Log deletion (repudiation), data delivery issues (repudiation), medical information disclosure (information disclosure), transaction privacy leakage, and wallet theft.
Vulnerabilities	Lack of privacy mechanisms.
Control and Countermeasures	Use privacy techniques, such as zero-knowledge proof; restrict access; and data encryption techniques.
**Connectivity**	
Assets/Components	Cloud and communication technologies.
Requirements	Access control, key management, trust management, and device/user authentication.
Threats	Data eavesdropping (information disclosure), side-channel attack (information disclosure), third-parties failures, communication modification (tampering), replay attack (tampering), and lack of input/output filtering in HIoT and APIs (tampering and information disclosure).
Vulnerabilities	Lack of encryption mechanisms in storage and all layers, insecure ecosystem interfaces, and unsecured network services.
Control and Countermeasures	Third-Party data distribution policy and monitoring and review of third-party services.
**HIoT**	
Assets/Components	HIoT devices, such MIoT and wearable.
Requirements	Localization, self-healing rearward and backward compatibility over the air programming/updating, and tamper-proof hardware.
Threats	HIoT type determination (information disclosure), HIoT tracking (information disclosure), battery-drain attack (denial of service), signal-jamming flooding (denial of service), maintenance compromise (elevation of privilege), device failure (tampering), and device tampering (tampering).
Vulnerabilities	Weak passwords, lack of HIoT device management, lack of physical protection measures, HIoT default settings, lack of HIoT device update mechanisms, lack of privacy mechanisms, unsecured interfaces, lack of authentication and authorization, and lack of/weak encryption.
Control and Countermeasures	Protect host and device security, authentication, and authorization.

## Data Availability

Data sharing not applicable.

## Appendix A. The Contributions of Included Studies in the SLR

**Table A1:** Contributions of HIoT security risk studies.

Title	Contributions
[S1] Risk Assessment Methodologies for the Internet of Medical Things: A Survey and Comparative Appraisal [12]	A literature review on HIoT risk, which investigates risk assessment methodology research and gives categorizations.
[S2] Threat Analysis for Wearable Health Devices and Environment Monitoring Internet of Things Integration System [91]	Wearable HIOT threat analysis using DREAD and STRIDE: assets and threats.
[S3] The Internet of Things for Health Care: A Comprehensive Survey [87]	A survey study on HIoT, including attacks taxonomy.
[S4] Internet of Things Security: A Review of Risks and Threats to Healthcare Sector [120]	Review on Health IOT security risks.
[S5] Security and Privacy in the Internet of Medical Things: Taxonomy and Risk Assessment [132]	Review study on HIoT showing risk assessment and attack taxonomy.
[S6] ISA Evaluation Framework for the Security of Internet of Health Things System Using AHP-TOPSIS Methods [124]	Risk assessment framework showing security requirements (i.e., 13 security evaluation attributes) and proposed Identified Security Attributes (ISA) framework used for decision making.
[S7] Security and Privacy for the Internet of Medical Things Enabled Healthcare Systems: A Survey [78]	A survey showing privacy and security requirements, threats, countermeasures in HIOT, authentication block diagram, and metrics for biometric authentication systems.
[S8] Review on security threats, vulnerabilities, and countermeasures of 5G enabled Internet-of-Medical-Things [149]	Review showing attacks and countermeasures in 5G HIoT, including BC applications.
[S9] Security and privacy risks for remote healthcare monitoring systems [89]	Review study showing vulnerabilities, security requirements, and countermeasures in HIoT.
[S10] Security Vulnerabilities, Attacks, Countermeasures, and Regulations of Networked Medical Devices—A Review [43]	Review study showing HIoT regulations, security requirements, vulnerabilities, and countermeasures.
[S11] Security in iomt communications: A survey [53]	Survey showing HIOT communication and security protocols.
[S12] A Survey on Security Threats and Countermeasures in Internet of Medical Things (IoMT) [104]	Survey showing HIoT security threats and countermeasures.
[S13] Privacy preservation in healthcare systems [99]	Review study showing HIoT privacy reservation taxonomy.
[S14] Review of Security and Privacy for the Internet of Medical Things (IoMT) [26]	Review study showing HIoT assets, security, and privacy.
[S15]Threat Modelling for Mobile Health Systems [92]	Threat modelling for mHealth using STRIDE and DREAD.
[S16] Data Risks Identification in Healthcare Sensor Networks [137]	Risk assessment for HIoT showing risks and countermeasures.
[S17] Data Protection and Privacy of the Internet of Healthcare Things (IoHTs) [29]	A review study covering Privacy by design for HIoT and privacy assessment recommendations.
[S18] Review of security challenges in healthcare internet of things [130]	Review showing security risk factor and countermeasures. Countermeasures are classified based on goals (preventive, detection, monitoring).
[S19] Security and Privacy in IoT–cloud-Based e-Health Systems—A Comprehensive Review [79]	Review study on HIoT showing privacy and security requirements based on layers.
[S20] Developing a comprehensive information security framework for mHealth: a detailed analysis [150]	Comprehensive security framework on mHealth taxonomy, showing the cloud-based hardware and software architecture and security requirements.
[S21] Security Benchmarks for Wearable Medical Things: Stakeholders-Centric Approach [126]	Benchmark framework showing 14 security and privacy attributes and metrics, which include authentication and access-control systems.
[S22] Authentication and Identity Management of IoHT Devices: Achievements, Challenges, and Future Directions [95]	Review study on IoT showing perception-layer security threats.
[S23] Device Security Assessment of Internet of Healthcare Things [147]	Risk assessment showing seven criteria to assist security.
[S24] Exploring Challenges and Opportunities in Cybersecurity Risk and Threat Communications Related to The Medical Internet of Things (MIoT) [136]	A survey showing the importance of engaging HIoT users in cybersecurity risk assessments.
[S25] IoMT-SAF: Internet of Medical Things Security Assessment Framework [118]	Risk assessment by design, a tool programmed using Python to allow users to evaluate security risks; web-based framework tests programmed 260 attributes divided into web–software–update–development life-cycle–storage–connectivity–trust–risk assessment–regulatory compliance–privacy–physical–intrusion prevention–memory protection–incident response–cloud service-authentication–access-control systems.
[S26] Privacy Risk Awareness in Wearables and the Internet of Things [90]	Privacy risk analysis by design, which proposes a privacy-risk-aware framework as a service. It is divided into four main components, one of which included privacy-risk metrics.
[S27] The internet of things in healthcare: an overview, challenges and model plan for security risks management process [148]	Security risk assessment based on ISO27005 with a case study in Kuala lumbur hospital.
[S28] The governance of safety and security risks in connected healthcare [80]	Review study showing the importance of merging safety with security in risk management, providing recommendations for cybersecurity governance.
[S29] Threat Modelling and Risk Analysis for Miniaturized Wireless Biomedical Devices [45]	Threat modelling conducted on MWBDs with a case study.
[S30] Security Assessment as a Service Cross-Layered System for the Adoption of Digital, Personalized and Trusted Healthcare [151]	Security risk assessment as service (by design); a layer of risk assessment in the system architecture.
[S31] Security Requirements of Internet of Things-Based Healthcare System: a Survey Study [102]	Review study showing cybersecurity and cyber resiliency requirements.
[S32] Towards Design and Development of a Data Security and Privacy Risk Management Framework for WBAN Based Healthcare Applications [93]	Security and privacy risk management framework for WBAN based on AAMI TIR57:2016 (Beta version after validation).

**Table A2:** Contributions of IdM security risk studies.

Title	Contributions
[S33] A Model for Privacy and Security Risks Analysis [152]	Categories of security risk factors mapped to security requirements.
[S34] Decentralized Identity Systems: Architecture, Challenges, Solutions and Future Directions [153]	Review study showing BC-IdM system architecture, components, and challenges.
[S35] A Comparative Study of Cyber Threats on Evolving Digital Identity Systems [94]	Threat model showing STRIDE model and BC-IdM requirement classifications.
[S36] A Digital Identity Stack to Improve Privacy in the IoT [145]	A structure model proposed for privacy of IdM systems.
[S37] A Survey on Blockchain-based Identity Management and Decentralized Privacy for Personal Data [154]	A survey showing BC-IdM evaluation with a focus on GDPR (right to be forgotten).
[S38] An Attack Tree Based Risk Analysis Method for Investigating Attacks and Facilitating Their Mitigations in Self-Sovereign Identity [116]	Security risk assessment to potential attacks in Self-Sovereign Identity (SSI). It identifies three security risks (fake identity–Id theft–DDoS), showing attack tree-based risk analysis models, including attack goals, vectors, and mitigations.
[S39] Analysis of Identity Management Systems Using Blockchain Technology [5]	Review and evaluation to uPort, Sovrin, and ShoCard BC-IdM systems, using the seven laws for digital identity.
[S40] Analyzing Interoperability and Portability Concepts for Self-Sovereign Identity [71]	Review and evaluation for interoperability and portability in BC-IdM systems.
[S41] Cloud identity management security issues and solutions: a taxonomy [155]	Review study showing a taxonomy of requirements and components of IdM systems.
[S42] Blockchain-Based Identity Management: A Survey From the Enterprise and Ecosystem Perspective [125]	Survey study identifying 73 Evaluation criteria for BC-IdM systems divided into three categories (compliance, end-user experience, technical).
[S43] Clear the Fog: Towards a Taxonomy of Self-Sovereign Identity Ecosystem Members [20]	GL study showing a taxonomy of key players in BC-IdM systems.
[S44] Criteria for evaluating the privacy protection level of Identity Management Services [123]	Survey showing evaluation criteria for privacy in IdM system life cycle.
[S45] Identity and access management in a cloud environment: Mechanisms and challenges [83]	Review study on IdM security threats in a cloud environment conducting security analysis.
[S46] Evaluation of Privacy and Security Risks Analysis Construct for Identity Management Systems [134]	Privacy and security risk analysis: evaluation of security taxonomy using the Delphi method.
[S47] Trust Requirements in Identity Management [103]	Review showing eight trust requirements in IdM systems and trust pillars (dependency, reliability, risk).
[S48] Identity Management as a target in cyberwar [84]	A review study showing three categories of the impacts of attacking IdM systems and attack vectors in IdM systems.
[S49] Introduction to Identity Management Risk Metrics [121]	Review study showing the importance of having IdM metrics. Metrics classified to Id Provider, provisioning, and identity metrics.
[S50] Measuring Identity and Access Management Performance - An Expert Survey on Possible Performance Indicators [156]	Survey study identifying 19 performance indicators (metrics) for IdM systems, which are categorized into five categories.
[S51] Cloud identity management: A survey on privacy strategies [85]	Survey showing a taxonomy for privacy features and properties in IdM systems.
[S52] A Metric-Based Approach to Assess Risk for "On Cloud" Federated Identity Management [86]	Review study showing risk metrics taxonomy for cloud-based federated IdM systems.
[S53] The Gaps of Identity Management in Fulfilling Personal Data Protection Regulations’ Requirements and Research Opportunities [33]	Survey showing the mapping of functional requirements from five data protection laws (GDPR, PDPA, PA1988, FIA, PIPA) to IdM system capabilities, and the importance of privacy impact assessments.
[S54] Privacy by Design in Federated Identity Management [101]	Survey study showing privacy principles, Privacy by Design RQMTS, and architectural RQMTS in federated IdM.
[S55] A Taxonomy of Privacy and Security Risks Contributing Factors [82]	Survey showing a taxonomy for privacy- and security-contributing factors in token-based IdM.
[S56] A new risk-based authentication management model oriented on user’s experience [22]	Risk assessment based on contextual data and user experience in authentication management.
[S57] The Seven Flaws of Identity Management: Usability and Security Challenges [135]	Survey showing seven challenges and considerations to compact IdM risks in IdM systems.
[S58] Trust Requirements in Identity Federation Topologies [157]	Review study showing service provider and Id provider risks and the need for trust requirements in FIdM systems.
[S59] User-Centric Identity Management: New Trends in Standardization and Regulation [21]	Review study showing the importance of complying with data protection laws in IdM systems and providing privacy.
[S60] Blended Identity: Pervasive IdM for Continuous Authentication [146]	Risk assessment for IDM in pervasive environment.

**Table A3:** Contributions of BC security risk studies.

Title	Contributions
[S61] Actor-based Risk Analysis for Blockchains in Smart Mobility [133]	Security risk analysis on smart mobility application based on public-permissioned BC.
[S62] Actor-based analysis Cyber-Security Risk Assessment Framework for Blockchains in Smart Mobility [138]	Cybersecurity risk assessment consisting of three steps with a case study on smart transportation.
[S63]Blockchain-based Application Security Risks:A Systematic Literature Review [158]	Review study showing security risks in Blockchain-based applications and countermeasures.
[S64] Exploring Sybil and Double-Spending Risks in Blockchain Systems [129]	Risk management for Sybil and double spending risks with a case study of Ethuerum-based healthcare systems.
[S65] Blockchain security risk assessment and the auditor [128]	Review study presenting an investigation on risks (four categories) in private Blockchain, with an emphasis on the importance of auditors’ role in risk assessments for BC applications.
[S66] The Blockchain Revolution: An Analysis of Regulation and Technology Related to Distributed Ledger Technologies [122]	A review study on Blockchain performance metrics and regulations.
[S67] A survey on Blockchain technology and its security [98]	A survey on security risks showing six risk categories, conducting Smart Contract bytecode analysis, and showing the need for BC regulations.
[S68] A Security Analysis of Blockchain-Based Did Services [88]	A survey on Destabilized identifiers (DID) security analysis, showing components, data flow, and 18 attack vectors divided to 7 main security threats.
[S69] Vision: A Critique of Immunity Passports and W3C Decentralized Identifier [131]	A review study on DID and Verifiable Credentials (VC) security analysis with a case study (COVID-19 immunity certificate system).
[S70] Analysis on the Privacy of DID Service Properties in the DID Document [32]	Review study showing security and privacy of DID, with a focus on DID document. It shows privacy breaches based on PIPA, and dataveillance privacy issues with potential countermeasures.
[S71] Quantum computers put Blockchain security at risk [159]	Survey on risks caused by quantum computing on Blockchain (forging digital signatures), showing the potential of using quantum computing as a countermeasure to prevent forgery.
[S72] A Security Risk Management Framework for Permissioned Blockchain Applications [140]	Security risk management for permissioned BC-based applications, which involves six-tier risk security framework with controls for every tier.
[S73] Security Threats of a Blockchain-Based Platform for Industry Ecosystems in the cloud [139]	Threat model showing security threats of BC-based IIoT, divided based on assets (IIoT, BC, and cloud), and showing the data flow diagram and risk metrics.
[S74] Air Gapped Wallet Schemes and Private Key Leakage in Permissioned BC Platforms [160]	Security risk analysis using Markov model to analyse the private key leakage risks in air-gapped wallet of permissioned BC and attack vector.
[S75] The Human-side of Emerging Technologies and Cyber Risk: A case analysis of Blockchain across different verticals [119]	A survey: interviews with leaders from financial companies resulted in five security risk categories for financial BC applications.
[S76] Risk Assessment of Blockchain Technology [141]	Security risk assessment using NISTSP-800-30, showing threats (4 types), related attacks, and potential countermeasures for attacks.
[S77] The Security Reference Architecture for Blockchains: Toward a Standardized Model for Studying Vulnerabilities, Threats, and Defenses [142]	Risk assessment model showing security architecture for BC and based on ISO/IEC15408, showing layers, assets, threat agents, vulnerabilities, threats, risks, countermeasures for every layer. IdM assets are covered.
[S78] Security and Privacy for Healthcare Blockchains [161]	A survey on privacy and security risks and requirements for BC-based medical data sharing systems (categorized into three health systems).
[S79] A Survey on Blockchain Technology: Evolution, Architecture and Security [162]	A survey showing an analysis of security risks of eight BC technologies, including vulnerabilities and threats, and their cryptographic techniques.
[S80] Alice in Blockchains: Surprising Security Pitfalls in PoW and PoS Blockchain Systems [163]	A survey study showing security risks in non-financial BC technologies, including databases, such as cocuhDB.
[S81] Detecting Blockchain Security Threats [143]	A survey study showing attack classifications in permissioned BC (i.e., Hyperledger Fabric), external threats/attackers, a four-control-classifications (including detective controls) BC security-monitoring pipeline, data flow, and evaluation metrics.
[S82] Attacking the trust machine: Developing an information systems research agenda for Blockchain cybersecurity [74]	Review study showing an investigation on BC security, which covers 5 common attack vectors (p2p network–consensus mechanisms–VM/language–application logic–client application/wallet), including 78 further attacks, and showing the users’, developers’, and attackers’ relations with BC applications and infrastructure.
[S83] Attacks and countermeasures on Blockchains: A survey from layering perspective [115]	Survey showing six-layered security framework (data–network–consensus–incentive–contract–application) and identifying potential attacks and countermeasures.
[S84] Security Assessment of Blockchain in Chinese Classified Protection of Cybersecurity [31]	Risk assessment for BC technologies (Bitcoin–Ethereum–Hyperledger Fabric), giving an evaluation for layers (P2P network–consensus–Distributed Ledger–contract), metrics, and controls based on Classified Protection Cybersecurity (CPC) law. The importance of BC being classified as critical infrastructure based on national standards, such as Chinese Classified Protection of Cybersecurity (CPC), to meet the country’s security requirements.
[S85] The Future of Cryptocurrency Blockchains in the Quantum Era [164]	Review study showing an analysis of the threat of quantum computing on cryptocurrency and BC, an analysis of cryptography techniques vulnerable to quantum, and countermeasures to quantum. It also shows quantum-safe and quantum-unsafe BC classifications.
[S86] Study on Security and Privacy-related Issues in Blockchain-Based Applications [165]	Review on the security and privacy threats and countermeasures in BC-based solutions.
[S87] Assessment of the Blockchain Technology Adoption for the Management of the Electronic Health Record Systems [81]	Literature review and assessment model showing use of Hierarchical Decision Model (HDM) methodology to investigate expert perspective on using BC in electronic health record (HER) management. Seventeen adoption-impacting factors are categorized into five categories (financial–social–technical–organizational–legal).
[S88] The Impact of Crypto-Currency Risks on the Use of Blockchain for Cloud Security and Privacy [166]	Review study showing the operational and market risks of using cryptocurrencies and their relation to the security and privacy of BC applications.
[S89] Potential Risks of Hyperledger Fabric Smart Contracts [167]	A survey showing 14 potential security risks related to the Smart Contracts that are written by Go language and used in Hyperledger Fabric BC; additionally, it proposes a tool to discover security risks in Smart Contracts.
[S90] Evil Chaincode: APT Attacks Based on Smart Contract [112]	Review study covering the advanced persistent threat (APT) attacks on Smart Contracts, which is an attack experiment on Hyperledger Fabric that provides recommendations to build countermeasures to security vulnerabilities in Smart Contracts.
[S91] Penetration testing framework for Smart Contract Blockchain [107]	A Review study and a penetration test for Smart Contracts, showing security threats and attack vectors in SC (categorized to network, application, data integrity, and end-user).
[S92] Systematic Review of Security Vulnerabilities in Ethereum Blockchain Smart Contract [108]	Review study on an Ethereum BC-based Smart Contract, showing vulnerabilities divided to three categories (solidity programming language–Ethereum virtual machine–Ethereum Blockchain design), SC security analysis tools, attacks, and preventive methods.
[S93] Smart Contract Security: A Software Lifecycle Perspective [109]	Review study on the security vulnerabilities in Ethereum Smart Contract and Hyperledger Fabric chaincode, which covers the Smart Contract life-cycle security model (with potential solutions).
[S94] On the Security and Privacy of Hyperledger Fabric: Challenges and Open Issues [168]	Review study showing the security threats and vulnerabilities toward architecture in Hyperledger Fabric divided into four layers (consensus–network–privacy–chaincode) and mitigation techniques.
[S95] Security Challenges and Opportunities for Smart Contracts in Internet of Things: A Survey [110]	Survey study and a threat model using STRIDE approach, showing security issues (inherited vulnerabilities–programming vulnerabilities–Attacks on SC), security audits for programming vulnerabilities (signature matching–formal verification–symbolic execution), and countermeasures.
[S96] Potential Risk Detection System of Hyperledger Fabric Smart Contract based on Static Analysis [111]	Risk analysis and transaction flows showing 16 potential security risks in SCs divided into three types of risks (non-determinism risk–logical security risk–privacy data security risk).
[S97] Blockchain Application in Healthcare Industry: Attacks and Countermeasures [113]	Review study identifying eight Attacks on BC applications in healthcare, as well as countermeasures.
[S98] The 51 Attack on Blockchains: A Mining Behaviour Study [105]	Review study covering an investigation of the 51 (the majority) attack risks on consensus mechanisms in Ethereum and Bitcoin BC technologies, showing characterization profile and anomalous behaviour to the suspicious miners and analysis of 10 prevention techniques.
[S99] The Internet of Things ecosystem: the Blockchain and privacy issues. The challenge for a global privacy standard [96]	Review study covering the seven privacy principles, privacy risks in IoT (identification–profiling–geolocation–liability for data breaches), the importance of DPIA and Privacy by Design in light of GDPR, and identifying the four main aspects to ensure the security and privacy of IoT applications.
[S100] Psychological and System-Related Barriers to Adopting Blockchain for Operations Management: An Artificial Neural Network Approach [1]	Survey (data collection) showing 178 responses from Malaysian manufacturing firms to investigate the barriers on BC adoption in operation management. Barriers are categorized into two main and seven secondary categories (psychological (information, usage, functional risks barriers), system-related (security and privacy, compatibility, interoperability, and system quality), showing that functional risks, security and privacy, and system usage showed the highest impacts on BC adoption.
[S101] Research on Progress of Blockchain Access Control [169]	Review on the functional requirements for BC-based access control, including an analysis of 16 BC technologies (Bitcoin, Ethereum, and consortium Blockchains)-based access control. It identified four main risks (privacy exposure issues, cross-organizational access issues, cross-chain access control problems, and performance optimization issues).
[S102] An empirical analysis of Blockchain cybersecurity incidents [170]	Security-incident analysis study that summarises and analyses 65 cybersecurity incidents related to Ethereum and Bitcoin BCs. Vulnerabilities are divided to Ethereum Virtual Machine bytecode, solidity, and BC network. Fraud and fraud victims classifications are given.
[S103]Evaluating Countermeasures for Verifying the Integrity of Ethereum Smart Contract Applications [127]	Review study including evaluation of the effectiveness of Ethereum Smart Contract vulnerability countermeasure solutions. A dynamic analysis tool to verify the integrity of SCs are proposed. A total of 11 static and dynamic countermeasures are identified and classified based on vulnerabilities and functionalities.
[S104] Security risk and response analysis of typical application architecture of information and communication Blockchain [144]	Review showing BC security risks, classified to storage layer (e.g., CouchDB and Level D.B.), protocol layer (consensus, security, and networking mechanisms), extension layer (SC, incentive, punishment mechanisms), and application layer (IoT-inherited vulnerabilities).
[S105] A Survey on Blockchain: Challenges, Attacks, Security, and Privacy [114]	Survey showing five security attack vectors in BC applications, as well as attacks and countermeasures. Seven Security and privacy (identity privacy and transaction privacy) requirements are identified.
[S106] The Risks of the Blockchain: A Review on Current Vulnerabilities and Attacks [117]	Review study identifying 24 security risks in BC, which are classified to four groups (BC structure vulnerabilities–consensus mechanism attacks–application attacks–attacks on the P2P network).
